# Heart Rate Variability and Perceived Recovery as Predictors of Performance in Athletes Competing in Sprint Events

**DOI:** 10.3390/s26061877

**Published:** 2026-03-17

**Authors:** Stefan Alecu, Gheorghe Adrian Onea

**Affiliations:** Department of Physical Education and Special Motricity, Faculty of Physical Education and Mountain Sports, Transilvania University, 500068 Brasov, Romania; onea.gheorghe@unitbv.ro

**Keywords:** heart rate variability, perceived recovery status, sprint performance, athlete monitoring, recovery assessment

## Abstract

Introduction: This study investigated heart rate variability (HRV) and perceived recovery status (PRS) in relation to sprint performance in competitive athletes involved in sprint events. A secondary aim was to explore potential gender-based differences in these relationships. Methods: Fifty-six sprint-trained athletes (21 males, 35 females; age 16–21) participated in a 5-day in-season microcycle. Daily morning HRV was measured using Polar H10 chest straps and the HRV4Training app, with the root mean square of successive differences (LnRMSSD) used as the primary HRV marker. Perceived recovery was assessed each morning using the PRS scale. On each day, athletes completed 20 m maximal sprint tests. Linear mixed-effects models were used to examine the relationships between LnRMSSD, PRS, gender, and sprint performance while accounting for repeated measurements within athletes. Results: Linear mixed-effects modeling revealed that LnRMSSD was a significant negative predictor of sprint time (β = −0.019, *p* = 0.003), indicating that higher parasympathetic activity was associated with faster sprint performance. PRS was also a significant negative predictor of sprint performance (β = −0.014, *p* = 0.008). Conclusions: Daily recovery markers were associated with sprint performance in competitive sprint athletes, with potential gender-specific patterns that should be interpreted cautiously. Both LnRMSSD and PRS were significantly associated with sprint performance, highlighting the relevance of combining physiological and subjective recovery markers in athlete monitoring.

## 1. Introduction

Maximizing sprint performance in competitive sport requires the careful balance of training stimulus and recovery status to optimize adaptation while minimizing the risk of fatigue-induced performance decrements. Sprinting over short distances, such as the 20 m sprint, is a fundamental component of most athletic disciplines and depends largely on neuromuscular power, central nervous system readiness, and efficient energy system engagement. Performance in these events is highly sensitive to acute fatigue and day-to-day fluctuations in readiness. Consequently, there is growing interest in practical, non-invasive tools that can reliably monitor recovery and predict sprint performance outcomes in high-level athletes.

Among the most widely adopted physiological tools for monitoring recovery is heart rate variability (HRV) [1,2,3,4,5], which reflects the dynamic balance between sympathetic and parasympathetic branches of the autonomic nervous system. HRV, and specifically the root mean square of successive differences (RMSSD), has emerged as a time-domain index of parasympathetic activity and recovery status [6,7,8,9]. The natural logarithm of RMSSD (LnRMSSD) is commonly used to normalize the distribution of this variable for statistical analysis [10]. Prior research has established that reductions in LnRMSSD are associated with high training load, incomplete recovery, and autonomic stress, whereas increases typically accompany rest, reduced training load, or parasympathetic rebound [11]. Consequently, HRV has become a cornerstone of athlete monitoring protocols, particularly in endurance- and sprint-trained athletes, where training stress accumulates across microcycles [12,13].

The use of HRV to predict sprint performance, specifically, however, remains underexplored. Heart rate variability (HRV) is widely used to monitor autonomic nervous system activity and recovery in athletes and has been proposed as a useful marker for training adaptation and fatigue monitoring [14,15,16]. While several studies have confirmed the sensitivity of HRV to fatigue and adaptation in intermittent and endurance modalities [17], relatively few have investigated its short-term predictive value in the context of explosive [18], maximal-effort actions such as sprinting [19]. Given the neuromuscular demands and central motor drive required for peak sprint performance, parasympathetic readiness—as indexed by HRV—may play a critical role in determining day-to-day performance fluctuations. Understanding whether morning HRV values are associated with same-day afternoon sprint performance may provide valuable information for athlete readiness assessments and training planning.

Complementary to objective physiological markers are subjective tools, such as the perceived recovery status (PRS) scale, which provide insight into an athlete’s self-perceived readiness for high-intensity exercise [20,21,22]. PRS is a single-item, 11-point scale ranging from 0 (very poorly recovered) to 10 (fully recovered) and is widely used in applied sport settings due to its simplicity and ease of integration into daily routines. Despite its simplicity, PRS has been shown to correlate with neuromuscular performance markers and training load and is responsive to changes in fatigue and recovery across microcycles. Subjective monitoring tools like PRS are capable of integrating multiple stress domains—including physiological, psychological, and emotional components—that may not be fully captured by objective measures such as HRV. The perceived recovery status (PRS) scale is a practical subjective tool used to evaluate athletes’ recovery state and has been shown to reflect fatigue and readiness to perform [21,23]. Importantly, the utility of subjective markers is often underestimated, yet evidence suggests they can outperform objective markers in certain contexts, particularly when monitored consistently and interpreted individually. Previous studies have reported associations between HRV indices and performance outcomes, suggesting that autonomic modulation may reflect athletes’ readiness to perform [14,24].

Despite the growing use of HRV and subjective recovery measures in athlete monitoring, limited research has examined the combined relationship between physiological (HRV) and perceptual (PRS) recovery markers with sprint performance, particularly in sprint-trained athletes. Furthermore, potential gender-related differences in these relationships remain insufficiently explored. Therefore, investigating these associations may provide valuable insights for optimizing training monitoring strategies. Recent research has also emphasized the potential influence of gender on recovery monitoring outcomes [25,26]. Male and female athletes exhibit different patterns of autonomic nervous system activity, hormonal fluctuations, and stress responses [27,28]. For example, HRV is known to vary across the menstrual cycle in female athletes due to shifts in estrogen and progesterone levels, which can affect vagal tone and the stability of parasympathetic indices [29]. These fluctuations may reduce the reliability of HRV as a short-term performance predictor in females, particularly when the menstrual cycle phase is not controlled. Conversely, subjective perceptions of recovery may provide a more consistent internal reflection of fatigue in female athletes, integrating both physical and hormonal influences. Despite these known physiological and perceptual differences, most studies investigating HRV or PRS have not examined potential gender-specific responses or predictive relationships in sprint-trained populations.

Daily HRV monitoring, particularly through short-term morning LnRMSSD recordings, has gained increasing attention as a practical tool for tracking autonomic fluctuations in response to training stress. Unlike single-point HRV assessments, daily monitoring captures within-athlete variability and provides insight into short-term changes in parasympathetic regulation across microcycles. LnRMSSD is considered especially suitable for day-to-day monitoring due to its relative robustness to recording duration and its sensitivity to acute fatigue and recovery status. Reductions in LnRMSSD have been associated with increased sympathetic dominance, elevated training stress, and impaired recovery, whereas stable or elevated values are typically observed under adequate recovery conditions.

From a performance perspective, sprinting relies heavily on neuromuscular efficiency, central motor drive, and rapid force production. Autonomic balance may influence these factors through interactions between central nervous system activation and peripheral recovery processes. Lower parasympathetic activity has been linked to increased physiological stress and reduced readiness, which may compromise explosive performance. Therefore, day-to-day fluctuations in LnRMSSD may reflect variations in central and peripheral readiness that are relevant for maximal sprint efforts.

Parasympathetic activity, indexed by LnRMSSD, has been associated with stress resilience, executive function, and central nervous system regulation. In high-intensity tasks such as sprinting, optimal performance requires efficient neural recruitment, rapid motor unit activation, and preserved neuromuscular transmission. Autonomic imbalance may reflect incomplete recovery at both systemic and neural levels. Consequently, monitoring daily LnRMSSD may offer indirect insight into central readiness and the capacity to produce maximal neuromuscular output during short-duration sprint tasks.

Therefore, the purpose of this study was to investigate the relationships between morning HRV (LnRMSSD), perceived recovery status (PRS), and 20 m sprint performance in competitive sprint-trained athletes over a 5-day in-season microcycle. A secondary objective was to explore whether these relationships differed between male and female athletes, in light of known gender-based differences in autonomic regulation and recovery perception. We hypothesized that LnRMSSD and PRS would be associated with sprint performance. Given previously reported sex-related differences in autonomic regulation and recovery perception, exploratory analyses were conducted to examine potential gender-specific patterns. However, menstrual cycle phase and hormonal status were not controlled, and therefore, gender-related interpretations should be considered exploratory.

## 2. Materials and Methods

### 2.1. Experimental Approach

The study was conducted between October 2024 and May 2025. The aim of this study was to determine whether heart rate variability (HRV), specifically the natural logarithm of the root mean square of successive differences (LnRMSSD), and subjective perceived recovery status (PRS) could predict fluctuations in sprint performance among trained sprinters during a short-term training cycle. To address this aim, we utilized a repeated-measures, observational design across a 5-day microcycle that reflected a typical in-season training schedule. This design was selected to capture day-to-day physiological and perceptual variations and their potential associations with sprint performance under real-world training conditions.

Athletes were not subjected to any imposed training intervention. Instead, they continued their normal training routines, which included both technical sprint drills and strength/power sessions, as prescribed by their club coaches. This ecologically valid design allowed for the examination of natural fluctuations in recovery and performance in a competitive environment without artificial manipulation. All measurements were scheduled and standardized to minimize confounding variables and improve replicability.

Each morning, immediately upon waking, athletes performed both HRV and PRS assessments in a rested and fasted state to ensure that the readings reflected true autonomic and subjective recovery. Afternoon sprint testing occurred at the same time each day (between 15:00 and 17:00), ensuring consistency in environmental and circadian influences on performance. The key dependent variable was 20 m sprint time, a commonly used and validated measure of short sprint ability. The independent variables included: Morning HRV (LnRMSSD), a physiological marker of parasympathetic activity and autonomic recovery, and morning PRS score, a validated subjective scale of recovery readiness.

By collecting these data on five consecutive days, we were able to capture within-subject variability in HRV, PRS, and sprint performance. The within-subject design reduces the influence of between-participant variability, increases statistical power, and aligns with recommendations for monitoring tools in elite sport contexts.

The theoretical underpinning of this study is that both physiological recovery (indexed by HRV) and psychological perception of readiness (indexed by PRS) reflect readiness to perform. Prior research has shown that HRV decreases in response to fatigue and stress, while PRS is sensitive to training load and correlates with neuromuscular performance markers. However, few studies have examined how both physiological and perceptual measures interact in predicting actual day-to-day performance outcomes in sprint-trained athletes.

This design allows us to address two critical questions in applied sports science:

Are HRV and PRS individually associated with fluctuations in sprint performance?

Can a combined model of HRV and PRS better predict performance outcomes than either marker alone?

The practical implications of this approach are significant. If either HRV or PRS can reliably anticipate decrements in sprint performance, they could be integrated into daily athlete monitoring to inform training modifications, tapering decisions, or injury risk reduction strategies.

This study aligns with the growing interest in using non-invasive, field-based monitoring tools to individualize training and recovery strategies in athletic environments, where logistical constraints often limit the use of laboratory-based performance assessments.

### 2.2. Participants

A total of 56 athletes competing in sprint events (21 males, 35 females; age range = 16–21 years; mean ± SD age = 18.2 ± 1.5 years) voluntarily participated in this study. All subjects were active sprint-specialized athletes representing four athletics clubs located in two major cities in Romania (Sibiu and Brașov). These athletes were exclusively involved in short-distance sprint events (60 m, 100 m, 200 m, and 400 m) and were engaged in structured training programs consisting of technical sprint work, strength training, and competition preparation under the supervision of certified coaches.

To be eligible for participation, athletes had to meet the following inclusion criteria: a minimum of 2 years of systematic sprint training experience, current participation in at least 4 training sessions per week, no history of neuromuscular injury or cardiovascular conditions in the past 6 months, no use of performance-enhancing substances or medications known to influence autonomic function, and willingness to comply with all daily testing procedures over a 5-day period. Athletes were excluded if they missed more than one day of data collection; reported illness, sleep disruption, or alcohol consumption within 24 h of testing; or demonstrated non-compliance with standardized HRV/PRS protocols (movement during HRV reading, delayed measurements).

Athletes were recruited through formal invitations sent to club directors and sprint coaches, followed by in-person information sessions where the purpose, requirements, and confidentiality measures of the study were explained. Parental or guardian consent was obtained for all participants under the age of 18, in addition to the athlete’s assent. Participants were assured that data would remain confidential, participation was voluntary, and they could withdraw from the study at any time without consequence.

All study procedures were approved by the Institutional Review Board of the Faculty of Physical Education and Mountain Sports under document no. 183/14.09.2024 and were conducted in accordance with the Declaration of Helsinki for human subjects research. The study was classified as minimal risk due to the non-invasive nature of the data collection methods (HRV and subjective scales), and participants were monitored by their coaches throughout the training cycle.

Of the total sample, 35 athletes regularly competed in the 60 m, 100 m, and 200 m events; 21 athletes specialized in the 400 m sprint, and the average training volume was 5.1 ± 1.2 sessions/week. All athletes were in a non-competitive in-season phase of their training during data collection. To minimize potential confounding variables related to gender, training phase, and event type, these characteristics were recorded and statistically accounted for during the analysis phase when needed. Two participants were excluded due to more than one invalid HRV recording (>5% artefacts), resulting in a final sample of 54 athletes.

### 2.3. Procedures

All testing was conducted across a 5-day in-season training microcycle, designed to represent typical training and recovery conditions for sprint-specialized athletes. Daily data collection included morning heart rate variability (HRV) and perceived recovery status (PRS), followed by afternoon sprint performance testing. Each athlete was tested under naturalistic training conditions in their own environment but with standardized instructions, equipment, and timing across clubs and locations.

Heart Rate Variability (HRV) Assessment: HRV was measured using the Polar H10 chest strap (Polar Electro Oy, Kempele, Finland), a device validated for accurate R–R interval detection in field-based sports research. The device was paired via Bluetooth with the HRV4Training mobile app (HRV4Training, Amsterdam, The Netherlands; version 3.8.7), which has been previously validated for RMSSD data acquisition.

HRV was recorded within 10 min of waking, before any food, caffeine, conversation, or physical movement. HRV was measured in a standardized seated position for all participants. Athletes were instructed to remain lying quietly on their backs with spontaneous breathing. The same body position was maintained across all five days, and no participant changed posture during the study period. Each measurement lasted 3 to 5 min, consistent with established standards for short-term HRV analysis. Athletes were instructed to conduct readings in a quiet, temperature-controlled room, minimizing ambient noise, light variability, or distractions. Athletes were asked to maintain regular sleep routines, refrain from alcohol or stimulant use, and avoid late-night physical activity during the study period. HRV4Training v. 3.8.7 was used to automatically calculate RMSSD values. These were exported daily and transformed using the natural logarithm (LnRMSSD) to account for non-normality.

Quality control protocols were implemented to check for ectopic beats or signal artifacts, and days with excessively noisy recordings (defined as >5% corrected R–R intervals) were excluded from analysis.

The root mean square of successive differences (RMSSD) represents short-term heart rate variability and is calculated based on the squared differences between consecutive R–R intervals—the time intervals between adjacent heartbeats. These intervals are measured in milliseconds and reflect the beat-to-beat variation in cardiac cycles. RMSSD (3) specifically captures the magnitude of variability between one heartbeat and the next, offering a reliable, time-domain index of parasympathetic (vagal) modulation of heart rate. RMSSD quantifies the short-term variability in heart rate by analyzing the differences between successive R–R intervals. It was calculated using the following formula:

$$RMSSD=\sqrt{\frac{1}{N-1}\sum {\left(i=1\right)}^{N-1}{\left(RR_{i+1}-RR_{i}\right)}^{2}}$$

where ${\mathrm{RR}}_{i}$ and ${\mathrm{RR}}_{i+1}$ are consecutive R–R intervals in milliseconds, and N is the total number of intervals in the measurement period.

Because RMSSD values are typically right-skewed, a natural logarithm (ln) transformation was applied to normalize the data distribution. This approach improves statistical validity in parametric analyses. LnRMSSD values were used in all inferential statistics, while raw RMSSD values were used for descriptive reporting and physiological interpretation.

Perceived Recovery Status (PRS) Assessment: Perceived recovery was assessed using the 0–10 PRS scale developed by Laurent et al. (2011) [21], which has been validated for monitoring subjective recovery status in athletic populations. A score of 0 indicates “very poorly recovered/extremely fatigued,” while 10 indicates “fully recovered/energetic.”

PRS was collected immediately after HRV assessment, before breakfast or training. Athletes were instructed to reflect on their entire body and mental state, rather than isolated soreness. The following standard prompt was used: “How recovered do you feel right now for high-intensity physical exercise?” Responses were recorded in a digital form (Google Forms, Google LLC, Mountain View, CA, USA). To ensure compliance, coaches supervised the completion of PRS scoring at each club location. PRS data were monitored daily to assess intra-athlete variation and detect potential outliers.

Sprint Performance Testing: Athletes completed 20 m maximal sprints daily as the primary performance outcome, selected due to its relevance in athletics and sensitivity to neuromuscular fatigue and recovery. The 20 m sprint was selected as a practical field-based measure of acceleration performance. Distances between 10 and 30 m are frequently used in sprint monitoring to evaluate early acceleration and neuromuscular performance, particularly during in-season periods when minimizing fatigue is important [30,31]. The 20 m distance allows assessment of both initial acceleration (0–10 m) and transition toward maximal velocity while maintaining a low testing load. All sprint testing was conducted in the afternoon between 15:00 and 17:00 to control for circadian variation. Testing was conducted on the same surface (outdoor synthetic track or indoor sprint track), consistent within each site across the 5 days. Athletes wore their regular training spikes or sprint shoes. A standardized warm-up protocol (10 min general mobility, 5 min sprint drills, and 3 × 20 m progressive sprints) was used before testing. Athletes began from a 3-point crouch stance behind the starting line. Sprint times were recorded using electronic dual-beam timing gates (Brower Timing Systems, Draper, UT, USA), positioned at the start line and 20 m mark. Each athlete completed 2 maximal effort sprints per day, with 3 min of passive recovery between trials. The fastest sprint time was retained for analysis to reflect peak performance capability.

Environmental conditions (temperature, wind, and track wetness) were logged daily to identify and account for performance-affecting variables. Athletes were monitored by club coaches throughout the testing period to ensure compliance with measurement timing, pre-test instructions, and safety protocols. To reduce variability, all measurements were taken at the same time each day, conducted with identical equipment and software, administered by the same personnel or thoroughly trained local assistants, and logged and reviewed daily for consistency and data quality.

The menstrual cycle phase and hormonal contraception use were not recorded during the data collection period. Consequently, no adjustment for menstrual phase-related fluctuations in autonomic activity was performed in the present analysis. Given the short 5-day monitoring window and the naturalistic design of the study, female athletes were assessed irrespective of cycle phase. Therefore, potential within-cycle hormonal influences on heart rate variability cannot be excluded.

### 2.4. Training Load Monitoring

During the 5-day microcycle, athletes followed their regular in-season training programs under coach supervision. Training content included technical sprint sessions, acceleration drills, and strength or power training. Session duration ranged between 75 and 110 min per day. Internal training load was quantified using the session rating of perceived exertion (sRPE) method. Athletes reported their perceived exertion approximately 30 min after each training session using the Borg CR-10 scale. Daily internal load was calculated by multiplying session duration (minutes) by the reported RPE score. Across the monitoring period, average session duration ranged between approximately 75 and 110 min, resulting in an estimated daily internal load between ~350 and ~700 arbitrary units depending on session intensity.

External training load variables were recorded by coaching staff and included the number of sprint repetitions, total sprint distance, and strength-training volume (sets and repetitions). Sprint sessions typically consisted of short acceleration and maximal velocity drills over distances between 20 and 120 m, with total sprint volume ranging approximately between 300 and 800 m per session, depending on the training day. Strength and power sessions included lower-body exercises such as squats, lunges, and plyometric drills performed with moderate-to-high intensity and low repetition ranges typical for sprint training.

Training content and structure were comparable across participating clubs and remained consistent throughout the 5-day monitoring period, with no planned taper or overload intervention introduced during data collection, which was carried out approximately 30 min after each session. Daily load was calculated as session duration multiplied by the RPE score. Mean weekly training frequency was 5.1 ± 1.2 sessions.

External load variables included the number of sprint repetitions, sprint distance volume, and strength training sets and repetitions, recorded by coaching staff. Training structure was consistent across clubs, and no taper or overload intervention was imposed during the monitoring period.

### 2.5. Statistical Analyses

All statistical analyses were conducted using SPSS Statistics (version 26, IBM Corp., Armonk, NY, USA). Because five daily measurements were nested within each athlete, linear mixed-effects models (LMM) were used to examine the predictive relationships between LnRMSSD, PRS, and sprint performance while accounting for the non-independence of observations. Athlete ID was included as a random intercept, and LnRMSSD, PRS, day, and gender were entered as fixed effects. Models were estimated using restricted maximum likelihood (REML). Statistical significance was set at *p* < 0.05. Given the elite nature of the sample, the number of participants reflects the available population of competitive sprint athletes involved in the monitored training period. Linear mixed models were used to account for repeated measurements within athletes. Degrees of freedom for the fixed effects were estimated using the Satterthwaite approximation.

### 2.6. Power Analyses

An a priori power analysis was conducted using G*Power statistical software (version 3.1.9.7, Heinrich Heine University Düsseldorf, Düsseldorf, Germany) with α = 0.05 and statistical power (1 − β) = 0.80 for detecting moderate effect sizes in repeated-measures designs. The analysis indicated that a minimum sample size of approximately 44 participants would be required to detect a moderate effect (f = 0.25). The final sample included 54 athletes, exceeding this threshold and indicating sufficient statistical power for detecting meaningful relationships between recovery markers and sprint performance.

## 3. Results

Descriptive statistics and group comparisons for LnRMSSD, perceived recovery status (PRS), and 20 m sprint performance are presented in Table 1. They are based on participant-level averages across the five-day monitoring period.

Descriptive comparisons indicated higher LnRMSSD and PRS values in male athletes and faster sprint times compared with female athletes. Male athletes demonstrated significantly higher LnRMSSD values compared to female athletes, indicating enhanced parasympathetic modulation and greater autonomic recovery status. Similarly, PRS scores were significantly higher in males (7.10 ± 1.40) than in females (5.90 ± 1.50; *p* = 0.022), suggesting more favorable subjective recovery perceptions among the male cohort. Regarding performance, 20 m sprint times were significantly faster in male athletes (3.04 ± 0.10 s) than in female athletes (3.26 ± 0.11 s; *p* < 0.001), consistent with known gender-based differences in sprint performance. Coefficients of variation (CV%) were below 10% for HRV and sprint time, indicating acceptable measurement stability, while higher variability in PRS (CV% > 19%) reflected the subjective and fluctuating nature of perceived recovery.

Together, these findings suggest that both physiological (LnRMSSD) and subjective (PRS) markers are not only distinguishable by gender but may also relate meaningfully to sprint performance differences in sprint-trained athletes.

As shown in Table 2, the linear mixed-effects model revealed that LnRMSSD was a significant negative predictor of sprint time (β = −0.019, SE = 0.006, t = −3.05, *p* = 0.003), indicating that higher parasympathetic activity was associated with faster sprint performance. PRS was also a significant negative predictor of sprint time (β = −0.014, SE = 0.005, t = −2.71, *p* = 0.008), demonstrating that higher perceived recovery corresponded to improved sprint outcomes.

A significant main effect of gender was observed (β = −0.21, SE = 0.04, *p* < 0.001), confirming faster sprint times in male athletes. Day also showed a significant fixed effect (*p* = 0.012), indicating performance fluctuations across the microcycle.

The random intercept variance attributable to Athlete ID was τ00 = 0.005, with a residual variance of σ^2^ = 0.004. The intraclass correlation coefficient (ICC = 0.56) indicated that 56% of the total variance in sprint performance was explained by between-athlete differences, supporting the use of mixed modeling.

### Data Quality and Reliability

A total of 280 HRV recordings were expected across the 5-day monitoring period (56 athletes × 5 days). Recordings exceeding 5% corrected R–R intervals were excluded prior to analysis according to the predefined quality control criterion. In total, 12 recordings (4.3%) were excluded. Detailed artifact percentage values were not retained in the archived dataset; therefore, gender-specific artifact rate comparisons could not be retrospectively calculated.

Inter-day reliability for LnRMSSD across the five consecutive days demonstrated good short-term stability (ICC = 0.84; 95% CI: 0.76–0.90; *p* < 0.001), supporting the consistency of HRV measurements under field conditions.

HRV recordings were conducted under spontaneous breathing conditions. Participants were instructed to remain seated and to maintain calm, regular breathing throughout the measurement period. Paced breathing was not imposed in order to preserve ecological validity.

## 4. Discussion

The purpose of this study was to evaluate the association between heart rate variability (LnRMSSD) [1,32,33,34] and perceived recovery status (PRS) [22,35] in explaining day-to-day fluctuations in sprint performance among trained sprint athletes. The main findings suggest that both HRV and PRS are significantly associated with sprint performance [29,30,36], though the strength of their associations differs between male and female athletes. These results support the integration of both physiological and perceptual recovery metrics in sprint athlete [30] monitoring and contribute to the growing body of literature emphasizing the multidimensional nature of fatigue and readiness in high-performance sport [37,38]. These findings indicate significant associations between daily LnRMSSD, PRS, and sprint performance. However, due to the observational design, the results should be interpreted as correlational rather than causal. Although autonomic balance and neuromuscular readiness may partly explain these associations, such mechanisms were not directly assessed and therefore remain speculative. Gender-specific findings should be interpreted with caution, as the menstrual cycle phase was not controlled and may have influenced HRV variability in female athletes. From a physiological perspective, the observed relationship between LnRMSSD and sprint performance in male athletes reinforces the role of parasympathetic regulation [39,40] as an important indicator of neuromuscular readiness [41]. LnRMSSD, as a time-domain HRV measure, reflects beat-to-beat variation in cardiac intervals mediated primarily by the vagus nerve. Higher LnRMSSD values are associated with enhanced parasympathetic activity and lower sympathetic dominance, which have been linked to improved recovery status, hormonal balance, and stress-buffering capacity. In the present study, the significant negative association between LnRMSSD and sprint time identified by the linear mixed-effects model suggests that when parasympathetic activity [42,43,44] is elevated, sprint performance tends to improve. This finding aligns with previous research showing that HRV is sensitive to both acute and chronic training loads and may serve as a practical tool for detecting readiness in explosive sports [45].

In contrast, the stronger predictive relationship observed between PRS and sprint performance in female athletes offers insight into the role of psychological and subjective self-awareness in managing fatigue and maintaining performance. Perceived recovery status, despite its simplicity, captures an athlete’s holistic sense of readiness—including physical, mental, emotional, and cognitive dimensions—which may not always align directly with physiological markers [46,47]. The significant negative association between perceived recovery status and sprint performance identified in the mixed-effects analysis suggests that athletes’ self-reported recovery perception was closely tied to actual neuromuscular output. These findings are in line with studies reporting that subjective wellness scores, including PRS, can be more sensitive than physiological measures in detecting subtle changes in fatigue, particularly in well-trained populations [26,48,49].

The observed differences between male and female athletes should be interpreted cautiously and considered exploratory, particularly because the menstrual cycle phase and hormonal status were not controlled in the present study. It is well-established that HRV is influenced by hormonal status, including fluctuations in estrogen and progesterone [50] levels throughout the menstrual cycle [51], which may introduce greater variability in female HRV measurements. This hormonal variability may reduce the reliability of HRV as a short-term performance predictor in females [52,53,54], especially when the menstrual cycle phase is not controlled. In contrast, subjective measures such as PRS may allow female athletes to self-integrate these fluctuations into their recovery perceptions, making PRS a more stable day-to-day indicator in this subgroup.

Another possible explanation for the gender-specific differences lies in differences in autonomic control and stress reactivity between males and females [55,56,57]. Research has shown that males typically exhibit greater vagal tone and larger HRV responses to training stimuli compared to females, potentially explaining why LnRMSSD was more predictive in the male cohort. Additionally, psychological coping mechanisms and reporting accuracy may differ between genders, with some evidence suggesting that female athletes are more reflective and consistent in reporting subjective ratings, which could account for the stronger association between PRS and performance in the female group.

From a training perspective, the observation that PRS and LnRMSSD were not always strongly correlated—yet both independently predicted performance—supports the idea that physiological and perceptual monitoring tools offer complementary information. While HRV captures autonomic regulation and the balance between recovery and stress, PRS may reflect the integration of multiple internal and external stressors [21], including muscle soreness, sleep quality, mental fatigue, and psychological readiness. This distinction is particularly important in the context of sprint events, which require maximal neuromuscular output and are highly sensitive to both physiological freshness and psychological engagement [58,59].

The results of the linear mixed-effects model indicated variability in sprint performance across the 5-day training microcycle. While HRV remained relatively stable across time, likely due to the athletes’ consistent training and recovery conditions, the fluctuations in PRS and sprint times suggest that these two variables are more sensitive to acute fatigue accumulation and readiness. The results suggest that male and female athletes may respond differently to training stimuli across the monitoring period. In addition to HRV and subjective recovery scales, several well-established physiological and neuromuscular markers have been used to monitor training stress and readiness. Biochemical indicators such as cortisol and creatine kinase (CK) have been shown to reflect endocrine stress responses and muscle damage, respectively, providing objective insight into accumulated load and recovery status [60]. While these markers offer valuable information, their assessment requires invasive sampling and laboratory analysis, which may limit daily applicability in field settings.

Neuromuscular performance tests, particularly the countermovement jump (CMJ), are also widely used as practical indicators of fatigue and readiness [61]. Reductions in CMJ height or power output have been associated with accumulated neuromuscular fatigue and performance decrements. CMJ testing provides a direct functional measure of explosive capability and may complement autonomic markers such as HRV by capturing mechanical output rather than cardiac regulation.

Together, biochemical markers, neuromuscular tests, and autonomic indices assess different dimensions of recovery. HRV reflects autonomic regulation, cortisol and CK reflect endocrine and muscular stress, and CMJ reflects neuromuscular output. Integrating these approaches may provide a more comprehensive profile of athlete readiness than relying on a single marker.

Overall, the findings of this study highlight the multidimensional nature of performance readiness in sprint-trained athletes. Monitoring only physiological markers or relying solely on subjective perceptions may provide an incomplete picture of an athlete’s ability to perform maximally on a given day. Instead, a more holistic monitoring approach that combines HRV and PRS and considers gender-specific trends may offer a more accurate and responsive system for managing performance and recovery in elite sprint athletes.

### 4.1. Limitations

A major limitation of this study is the lack of menstrual cycle phase and hormonal contraception tracking in female athletes. Because HRV fluctuates across the menstrual cycle due to hormonal changes, uncontrolled phase variation may have increased LnRMSSD variability in females and weakened HRV–performance associations. As a result, the observed gender differences, including the stronger predictive value of PRS in females, may reflect measurement instability rather than true sex-based differences. Therefore, conclusions regarding gender comparisons should be interpreted with caution.

Although recordings exceeding 5% corrected R–R intervals were excluded, detailed artifact percentage data were not retained for secondary analysis. Consequently, gender-specific comparisons of artifact rates could not be performed. Future studies should archive artifact metrics systematically to enhance methodological transparency.

Therefore, the gender-related findings reported in this study should be considered exploratory and hypothesis-generating rather than confirmatory.

### 4.2. Practical Applications

The findings of this study support the integration of both physiological and subjective monitoring tools into daily athlete management systems for sprint-trained individuals. Heart rate variability, specifically LnRMSSD, can serve as a practical and objective indicator of autonomic recovery and readiness to perform. Perceived recovery status (PRS) may also provide a useful and accessible indicator of perceived readiness and recovery status in sprint athletes. Coaches and practitioners are encouraged to adopt a dual-monitoring approach that incorporates both HRV and PRS to capture a more comprehensive profile of athlete readiness. These tools can be easily implemented using widely available technology, and they require minimal resources and provide actionable feedback within minutes. Daily monitoring using HRV and PRS may assist in identifying early signs of fatigue, informing individualized training adjustments, and optimizing performance outcomes across microcycles.

In the present model, gender was included as a fixed effect; however, interaction effects were not tested. Therefore, the results do not allow conclusions regarding differences in the strength of associations between male and female athletes. Practitioners should be cautious about interpreting HRV values in isolation, particularly in female athletes, and consider subjective indicators as a valid and potentially more stable signal of readiness. By combining objective and subjective data streams, support staff can make more informed, athlete-centered decisions around training load, recovery needs, and competition preparation.

## 5. Conclusions

The findings demonstrate that daily recovery markers are significantly associated with sprint performance in competitive sprint athletes. Both heart rate variability, expressed as LnRMSSD, and perceived recovery status demonstrate meaningful associations with sprint performance for 20 m sprint outcomes across a short in-season microcycle. Higher LnRMSSD and higher PRS scores correspond to faster sprint times, supporting the relevance of autonomic and perceptual recovery monitoring in sprint settings.

Potential gender-related trends were observed; however, these findings should be interpreted as exploratory due to the lack of control for menstrual cycle phase and hormonal influences. LnRMSSD showed a stronger association in male athletes, whereas PRS emerged as the more consistent predictor in female athletes. These differences suggest that physiological and subjective indicators may reflect recovery and readiness through different mechanisms across sexes, reinforcing the need for individualized interpretation.

Daily LnRMSSD and perceived recovery status were significantly associated with sprint performance across the five-day monitoring period. These findings support the potential practical value of combined physiological and subjective monitoring. However, conclusions remain limited to associative interpretation, and longitudinal studies with tighter physiological control are required to confirm their predictive utility.

Overall, the combined assessment of HRV and PRS offers a practical approach for identifying day-to-day fluctuations in readiness and performance. Integrating both objective and subjective measures may enhance training load management, optimize recovery strategies, and support performance maintenance in sprint-trained populations.

## Figures and Tables

**Table 1:** Descriptive statistics for LnRMSSD, PRS, and sprint time.

Variable	Gender	Min	Max	X	SD	St.Err	CV%	CI95%		*p*
								Lower	Upper	
LnRMSSD (AU) *	M	3.35	4.45	3.90	0.27	0.059	6.92	3.78	4.02	0.019
	F	3.00	4.25	3.62	0.30	0.051	8.29	3.52	3.72	
PRS (0–10 scale)	M	5.00	9.00	7.10	1.40	0.306	19.72	6.47	7.73	0.022
	F	3.50	8.50	5.90	1.50	0.254	25.42	5.39	6.41	
Sprint Time (s)	M	2.83	3.25	3.04	0.10	0.022	3.29	3.00	3.08	<0.001
	F	3.02	3.55	3.26	0.11	0.019	3.37	3.22	3.30	

Note: Min = minimum; Max = maximum; X—mean; SD = standard deviation; St.Err = standard error; CV = coefficient of variation; CI95 = confidence interval; *p* = significance threshold. * AU = arbitrary units. LnRMSSD values are reported in arbitrary units (AU) to reflect the log-transformed, unitless nature of the data. Values represent participant-level means averaged across the five consecutive days for each athlete. Group statistics are calculated using these individual mean values.

**Table 2:** Linear mixed-effects model predicting 20 m sprint time.

Effect Type	Predictor/Component	Estimate (β) *	SE	t	*p*	Variance
Fixed Effect	LnRMSSD	−0.019	0.006	−3.05	0.003	—
	PRS	−0.014	0.005	−2.71	0.008	—
	Gender (Male vs. Female)	−0.210	0.040	−5.32	<0.001	—
	Day	—	—	—	0.012	—
Random Effect	Intercept (Athlete ID)	—	—	—	—	0.005
	Residual	—	—	—	—	0.004

Intraclass correlation coefficient (ICC) = 0.56. Note. * β represents unstandardized regression coefficients. SE = standard error. Gender coded as 0 = Female, 1 = Male. Negative β values indicate faster sprint times with higher predictor values. The linear mixed-effects model was estimated using all daily observations (five measurements per athlete). Athlete ID was included as a random intercept to account for repeated measurements. No averaging across days was performed for this analysis.

## Data Availability

The original contributions presented in this study are included in the article. Further inquiries can be directed to the corresponding author.
